# Safety and Parental Satisfaction With Early Discharge of Preterm Infants on Nasogastric Tube Feeding and Outpatient Clinic Follow-Up

**DOI:** 10.3389/fped.2020.00505

**Published:** 2020-08-25

**Authors:** Rahel Schuler, Harald Ehrhardt, Walter A. Mihatsch

**Affiliations:** ^1^Department of General Pediatrics and Neonatology, Justus Liebig University, Giessen, Germany; ^2^Hospital Pforzheim, Teaching Hospital of Heidelberg University, Pforzheim, Germany; ^3^Department of Pediatrics, Ulm University, Ulm, Germany; ^4^University of Applied Sciences, Neu-Ulm, Germany

**Keywords:** preterm, early-discharge, tube-feeding, parental satisfaction, outpatient clinic follow-up

## Abstract

**Aim:** To evaluate our early discharge program of preterm infants with nasogastric tube feeding (NTF) and close outpatient clinic follow-up with regard to safety, parent satisfaction and parental stress level.

**Methods:** 119 preterm infants were discharged on NTF from our tertiary care neonatal unit (median gestational age 31.0 weeks, median birthweight 1,650 g). Parental satisfaction was evaluated by a standardized questionnaire. For safety assessment growth until term equivalent age and re-hospitalizations within 2 months after discharge were evaluated.

**Results:** Infants were discharged home at a median gestational age of 35.4 weeks after a median hospital stay of 22 days. Follow up was attained in 95 of 104 parent-infant dyads. The majority of parents (94%) reported that they had made the right decision in taking their infant home on NTF. At the time of discharge 86% of parents felt very well-prepared to perform NTF. 70% Of parents rated their stress level at home as low (≤2 out of 5). There were no NTF associated readmissions and no growth faltering until term equivalent age.

**Conclusion:** Early discharge of preterm infants with NTF together with outpatient clinic follow-up is very well-accepted by parents and appears to be safe.

## Introduction

Hospitalization of preterm infants easily reaches several weeks or months. This is a very stressful time for parents, siblings and the infant and may have long-lasting negative psychological effects (1). Historically, preterm infants have been discharged after reaching a certain week of gestation or a certain body weight (2, 3). The policy statements of the AAP of 1998 and 2008 recommended a more physiological approach (2). Three physiological competencies that are generally recognized as essential before discharge are oral feeding sufficient to support appropriate growth, the ability to maintain normal body temperature in a home environment, and sufficiently mature respiratory control (4, 5). Although interrelated, not all competencies are achieved by the same postnatal age in a given infant (3). Some preterm infants have a period of physiological stability toward the end of the hospital stay but still need nasogastric tube feeding (NTF). Feeding related difficulties are an often-cited reason for hospitalization beyond 36 weeks postmenstrual age (6). During these final weeks parents and siblings frequently experience frustration and feel burdened (7, 8). To reduce the time of hospitalization early discharge programs have been established. Early discharge with follow up by a home care nurse specialist after reaching physiological competencies regardless of weight has been shown to be safe in VLBW infants on full oral feeds (4). To further facilitate early discharge in preterm infants home NTF may be the key intervention (9). In preterm infants systematic discharge on NTF with follow up by a home care nurse specialist has been suggested to be safe, improve breast-feeding rates, and result in no impairment of weight gain (7, 8, 10, 11), however more data is still needed (9). In addition, the risk of nosocomial infections may be reduced (7, 9).

In Europe, discharge of preterm infants on NTF is uncommon. In several European regions such as our region there is a structural lack of pediatric home care nursing and frequently routine home care nursing cannot be offered to preterm infants after discharge. Prompted by our parents and due to the existing evidence of the benefits of early discharge (7, 8, 10, 11) a new approach of early discharge on NTF with close hospital outpatient clinic follow up instead of home care nursing was established at our unit.

The aim of the present retrospective study was to evaluate this new home NTF program with regard to safety, parental stress level and parent satisfaction, using a standardized questionnaire.

## Materials and Methods

### Patients

All inborn preterm infants who were discharged on NTF from Pforzheim children's hospital in 2017 and 2018 were eligible for the present study. Pforzheim hospital, 75175 Pforzheim, Germany is a regional tertiary care teaching hospital of Heidelberg University, Germany caring for about 3,500 births per year. It is the only pediatric hospital in the region.

### Intervention

Up to October 2016 preterm infants were discharged after the establishment of full oral feeds. Mothers stayed in hospital with their baby at best 1 or 2 days before discharge.

As of the end of 2016, gradual implementation of a more family integrated care approach was started helping parents cope in the NICU (1). Our basic massage was “You are a good and competent parent,” Carefully coordinated discharge planning together with the families was performed starting early after admission. One important step was that preterm infants were transferred to the neonatal step-down unit right after the achievement of respiratory stability, which was defined as no need for non-invasive ventilation such as CPAP or High Flow Nasal Cannula. Rooming-in was strongly encouraged and there was unrestricted 24/7 presence of parents. The rooms were double rooms with two parent-infant-dyads per room and an attached bathroom. Families were assigned one physician and her representative. Parents were continuously supported and empowered to develop competences in caregiving activities, decision-making and active problem solving. They were instructed by the professional team and took over more and more responsibility in caring for their child. They were trained to administer oral medications, tube, bottle, and nipple feedings. Tube placement and administration of IV medication was done by nurses only. If accidental tube displacement occurred at home replacement of the tube was done in an outpatient setting either at the hospital or the local pediatrician. Parents received specific information and training about important issues when caring for their infant at home, such as signs of infant illness and how to handle emergency situations, feeding tube displacement and problems with checking the tube position.

Discharge criteria independent of corrected age were as follows:

- Respiratory stability on room air or supplemental oxygen via nasal cannula
- Absence of apnea and bradycardia requiring stimulation - either stable on caffeine with a home monitor or stable without caffeine
- Ability to maintain a body temperature >36.5°C
- Adequate enteral fluid intake (at least 150 ml/kg/d) via nasogastric tube
- Ability to partly (>50%) feed via oral route – to prevent hypoglycemia in e.g., nocturnal feeding tube displacement
- Sustained pattern of weight gain
- Optional discharge on NTF
- Parents confident in intermittent gentle bolus NTF using syringes, administration of oral medication, and use of home monitor (perfusion pumps were not used)
- Health care team is convinced that parents are able to perform NTF safely and are capable of caring for their preterm child at home
- Parents judge themselves confident with home NTF

Scheduled multidisciplinary developmental follow up at the associated developmental care center at corrected age of 3 months, 6 months, one and two years was provided.

Scheduled close follow-up at the hospital preterm infant outpatient clinic, first visit within 1 week after discharge and thereafter every 7–14 days until independent feeding and weight gain without falling off percentiles were established. Additionally, telephone service (24/7) was provided by the team of the neonatal intensive care unit.

### Data Acquisition

**Figure 1:**
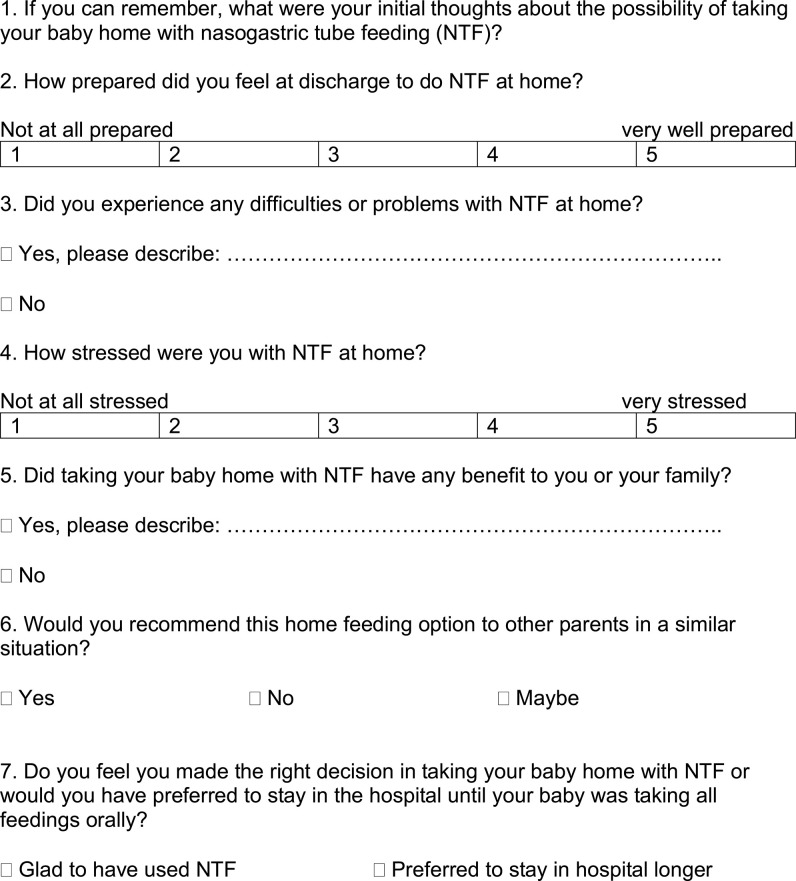
Questionnaire, translated into English language.

To systematically assess parent satisfaction and stress levels and to ensure comparability with the literature, the study was based on a previously published questionnaire (11), which was translated and adapted to the German language (Figure 1; one questionnaire per family) (German version as Supplement). Parents were asked about their initial feelings on taking the infant home with NTF, problems with NTF at home, benefits for the family, recommendations for other families and whether they feel they made the right decision or not. They were asked to rate their preparedness regarding NTF and their stress level with NTF at home on a Likert-type scale from 1 to 5. The questionnaire was either sent to parents by mail or handed out at the outpatient clinic during a scheduled visit. Telephone interviews were conducted if the questionnaire was not sent back via mail.

For safety assessment, weight gain after discharge until term equivalent age and readmissions within the first two months after discharge were analyzed. Clinical characteristics such as birth weight, postmenstrual age, or information on readmission were extracted from the hospital database. Parents were also asked about readmissions. Anthropometric data were standardized using the Fenton growth chart (12).

### Ethical Aspects

The study has been approved by the Medical Ethics Review Board of the National Baden-Wuerttemberg Medical Association, 70597 Stuttgart, Germany, F-2018-063, 30.07.2018. Informed parental consent was obtained for all children included in the study.

### Data Analysis

Results are presented as median [interquartile range (IQR)] or as percentage as appropriate. Free comments are presented in categories. Occasionally parents did not provide an answer to a single question. The number of missing answers for questions 1–7 were 0, 3, 4, 6, 9, 5, and 6. Therefore, calculation of percentages was based on the number of retrieved answers. The associations between length of rooming-in and the mode of answering the questionnaire (by using the written questionnaire or by telephone interview) on the stress level with NTF at home were analyzed by Spearman's rank correlation and chi-square test. Data was analyzed using Microsoft Excel (Microsoft Corporation, One Microsoft Way, Redmond, WA 98052-6399, USA).

## Results

One hundred nineteen infants (77 male, 53 female) of 104 parent-child dyads were discharged home with NTF. There were 90 singletons, 13 twins and one triplet. The clinical characteristics of the infants at birth, discharge, and the last visit near term equivalent age are given in Table 1. With regard to NEC, moderate or severe BPD, severe IVH (stage 3–4), and ROP, we did not experience a single case of Bell stage >2 NEC since 2016. There was one infant (22 weeks of gestation) with BPD, severe IVH and stage 3 ROP and one additional case of stage 3 ROP (23 weeks of gestation). Seventy-two infants were still dependent on caffeine for respiratory stability and were discharged with home monitor.

**Table 1:** Clinical characteristics data is given as median (interquartile range).

	**Birth**	**Discharge**	**Last measurement**
N	119	119	112
Gestational age (wks)	31.0 (29–33.5)	35.4 (34.5–36.7)	38.6 (36.9–40.1)
Weight (g)	1,650 (1,260–2,050)	2,190 (1,975–2,435)	2,705 (2,438–3,063)
z- score	−0.1 (−0.8 to 0.4)	−0.7 (−1.5 to −0.3)	−0.8 (−1.5 to −0.1)
HC (cm)	29.5 (27.5–31)	31.0 (30.0–32.0)	33.0 (32.0–35.0)
z-score	0.3 (−0.2 to 1.0)	−0.5 (−1.4 to 0.1)	−0.1 (−0.9 to 0.2)

All 104 parent-child dyads were approached. The overall response rate was 91% (95 retrieved questionnaires); 8 families could not be contacted via mail or telephone; 1 family was not able to answer in German language. There was no significant difference in the stress level of the parents which used the questionnaire or which were approached by telephone interview. Initially, when informed about the option to take their infant home with NTF, parents described their feelings as joy and relief (49%), as anxiety and worry (44%), and some expressed both feelings at the same time (7%). Retrospectively, 89% of the parents (82 of 92) rated their preparedness to tube feed their infant at the time of discharge as 4 or 5 out of 5 (very well-prepared). 13% Of the parents (12 of 91) mentioned difficulties with home NTF such as the tube being accidentally pulled out (*n* = 8) and problems with checking the position of the tube via aspiration (*n* = 8). Mean stress level regarding NTF at home was low (2.0 out of 5), 64 of 89 (72%) of the parents rated their stress level as low (1 or 2 out of 5), 13% (12 of 89) rated their stress level as high (4 or 5 out of 5).

The association of length of rooming-in with stress level, preparedness and right decision is given in Table 2. Overall the median length of hospital stay was 22 days (13 to 38) including 8 (4–14) days of rooming-in. The majority of the 13 parents who decided against rooming-in reported that they were well-prepared (92%, 12 of 13), were still happy with their decision of early discharge on NTF (10 of 13; 77%), and experienced low stress levels (1–3) at home (10 of 13; 77%). Over all the stress level with NTF at home significantly decreased with increasing length of rooming-in (Spearman's rank correlation test, *p* < 0.05). One singleton mother with severe post-traumatic stress disorder, anxiety, and mild depression after migration experiences, had the longest period of rooming-in (35 days). Her infant did fine, she felt very well-prepared, and was happy with taking her infant home on NTF, yet she did report of high stress levels. Due to the post- traumatic stress disorder after migration she was excluded from further analysis. Significantly more parents 20% (95% CI 10–32%; 9 of 46) with no or up to 1 week of rooming-in experienced stress (stress level 4–5) with NTF at home than parents with longer periods of rooming-in 4% (95% CI 1–14%; 2 of 48; chi-square test *p* < 0.05). The majority of parents (79 of 86, 92%) felt that taking their infant home with NTF was beneficial. The most frequently reported benefits were being at home and experiencing normal life together as a family. Four parents (5%, 4 of 90) would not recommend NTF at home to other parents albeit two of them still felt taking their own baby home on NTF was the right decision for themselves. The gestational ages of the infants were 23.4, 34.9, 35, and 35.9 weeks. No NTF associated complications such as aspiration, aspiration associated pneumonia, pneumonia, or bronchial administration of feed were reported. One infant who suffered from severe growth retardation and an unknown genetic defect died 3 days after discharge from the genetic defect.

**Table 2:** Association of length of rooming-in with stress level, preparedness, and right decision.

**Length of rooming-in (days)**	**Hospitalization (days)**	***n***	**Stress level 4–5**	**Preparedness Score 4–5**	**Right decision**
0	10 (6–13)	13	23%	92%	77%
1–7	13 (10 – 24)	33	18%	91%	100%
8–14	19 (14 – 36)	30	7%	87%	97%
15-21	34 (26 – 45)	8	0	88%	100%
22–27	41 (34 – 58)	8	0	88%	88%
29–35	47, 50, 66	3	33%	100%	100%

*Days of hospitalization are given as median (IQR)*.

Five Parents answered that they should have stayed in hospital longer whereas, the majority of parents (94%, 84 of 89) reported that taking their baby home on NTF was the right decision. These 5 parents and 22% of the parents who were happy with early discharge were stressed at home with NTF (stress levels 3–5). The length of hospitalization decreased with increasing gestational age at birth. Even very preterm infants (<26 weeks of gestation) were discharged well-before their due date (Table 3).

**Table 3:** Gestational age at birth, median (IQR) corrected gestational age at discharge, and median length of stay (IQR).

**Gestational age at birth (weeks)**	**Corrected gestational age at discharge (weeks)**	**Length of stay (days)**	***n***
22–26	36.0 (34.0–38.3)	80 (62–89)	8
27–31	34.7 (34.3–36.0)	37 (26–45)	52
32–36	35.6 (35.0–36.0)	14 (9–16)	59

All infants but one (22 weeks of gestation, neurological impairment after severe IVH, BPD, and stage 3 ROP) were weaned at home to full oral feeding. Data on the time of removal of the tube was recorded in 2018 only and is available for 47 of 70 (67%) infants. Removal of the tube was at 36.9 (36–37.6) weeks PMA, after a median time of 8 days (3–14).

Within 2 months after discharge, 30 infants were rehospitalized (Table 4), 3 infants were rehospitalized twice (33 rehospitalizations). The reasons for unplanned rehospitalization (*n* = 22) were constipation, gastroesophageal reflux, fussiness, viral infections, apnea after vaccination and bacterial infection; for planned rehospitalization (*n* = 11) vaccination, inguinal hernia repair, initiation of propranolol/ganciclovir therapy, and discontinuation of home monitoring. No rehospitalization was related to NTF difficulties. Ninety five percentage of parents whose infant was rehospitalized and 94% of those not rehospitalized felt they had made the right decision regarding early discharge with NTF.

**Table 4:** Characteristics of rehospitalized and not rehospitalized infants.

	**Rehospitalized**	**Not rehospitalized**
***N***	**30**	**89**
Gestational age (weeks)	29.9 (28.6–32.8)	32.3 (30.6–34.3)
Birthweight (g)	1,325 (980–1,788)	1,740 (1,420–2,160)
GA at discharge (weeks)	36.3 (34.5–37.9)	35.4 (34.6–36.3)
Length of hospital stay (days)	37 (24–52)	17 (10–30)
Length of rooming-in (days)	11.5 (8–16)	8 (4–13)

*Data is given as median (IQR)*.

## Discussion

This is the first study evaluating an early discharge program without home care nursing. Early discharge of preterm infants on NTF with regular close outpatient clinic follow up instead of home care nursing was safe and parental satisfaction was high. In addition, this is the largest study on parental satisfaction with discharge of preterm infants on nasogastric tube feeding (10, 11). The follow up of 91% was considerably higher than in previous studies (67 and 67%) (10, 11). In line with previous studies with home care nursing (10, 11), our study reconfirms that overall satisfaction with early discharge on NTF is very high. The overwhelming majority of parents felt they had made the right decision and most would recommend it to other parents. In accordance with the other studies, parents ranked their overall level of preparedness to perform NTF at home as high (9–11).

Parents in our study had a low median stress level regarding NTF (2 out of 5). Surprisingly, the proportion of parents who did not feel stressed at home (stress level 1–2 out of 5) was the same as in the Sturm cohort (70%) despite the major difference that we could not offer a home nursing program (11). Achieving such a low overall stress level in our cohort even without a neonatal home visiting nurse is most likely due to the rooming-in design and systematic supporting, empowering and teaching parents in the care, and early sharing responsibility for the well-being of the child. A hospital-based neonatal home care nursing program may further reduce parenteral stress.

The discharge program was embedded in the concept of family integrated care and rooming-in (13). Rooming-in for 8–14 days was associated with lower stress levels at home than shorter periods (0–7 days) (Table 2) supporting this approach. Even though parents did feel more stressed after a shorter duration of rooming-in, they still felt well-prepared and felt they had made the right decision to go home with NTF. Altogether, the data suggest that at least a week of rooming-in, training and instruction may be required to gain confidence. Unfortunately, other studies did not report on family integration or rooming in (10, 11).

Early discharge on NTF appeared to be safe. No adverse events or NTF related re-hospitalizations were reported. Similar to a previous study (14) there was no growth faltering till term equivalent age, head growth even showed catch-up growth (Table 1). Weaning from tube feeding took on average 8 days similar to a previous study (10).

Hospital readmission rate (26%) in our cohort was slightly higher than those of other early discharge programs [11.6% (14), 20% (7), 10% (5), 12% (10)] albeit gestational age at discharge was similar. The readmission was not related to NTF. However, readmission was related to physiological immaturity. Home care nursing may well have prevented some of the readmissions for e.g., constipation (*n* = 3), gastrointestinal reflux (*n* = 3), or fussiness (*n* = 3). Of note, even the parents of re-hospitalized infants (*n* = 30) were overall satisfied and only two did regret their decision of discharge with NTF.

Preterm infants of < 32 weeks gestational age were discharged at 35.4 (34.5–36.7) weeks, where it is common practice in Germany to discharge these infants at about 38 weeks (15, 16). The preterm infants of the present study were discharged also on average 2 weeks earlier than a recent population of preterm infants of the English Neonatal Research Database (17). Therefore, beyond the advantages for infants and parents, early discharge on NTF may also be beneficial regarding admission capacity (10) and could be advantageous from an economical point of view.

In our cohort, infants of 27–31 + 6 weeks of gestation were discharged at 34.7 (34.3–36.0) weeks (Table 3). In the literature some subgroups of VLBW infants achieved full enteral feeding on average at about 35 weeks (6, 18–20). On average, these infants were discharged beyond 36 weeks of gestation. These data suggest that it may have been possible in our infants to push feeding advancement at the cost of a longer hospital stay. Parents decided that being at home was their immediate priority even at the cost of readmission later for clinical routine procedures such as vaccinations, inguinal hernia repair or changes in pharmacotherapy. They took over the responsibility for achieving full enteral feeding.

Besides loss of follow up (9%) and recall bias the main limitation of the present retrospective study is the lack of a control group to compare parental satisfaction, stress level after discharge, and growth parameters e.g., in a matched pairs analysis. In one prospective randomized clinical trial without home NTF, Saenz et al. have shown that parental anxiety was not different between an early and a standard discharge group. The well-being scores of parents in the early discharge group were consistently higher from discharge to 3 months after discharge and maternal depression was significantly lower in the early discharge group (21). Situational anxiety was lower in the early discharge group on NTF in the study of Ortenstrand et al. (22). We therefore speculate that in our setting stress levels would be higher in a standard discharge group as early discharge facilitates a quicker establishment of normal family life (21) as mentioned by many of the parents in our cohort as the benefit of early discharge.

In summary early discharge with NTF together with close outpatient clinic follow-up or home care nursing appears to be safe (7, 10), has substantial benefits for the family infant relationship (23), and is very well-accepted by parents. Future randomized clinical trials should, therefore, further evaluate early discharge with tube feeding. The influence of optimizing home care and its effect on parental stress levels as well as the comparison of overall stress levels of parents of infants discharged early with tube feeding and those discharged according to standard care are important fields of future research. Long-term evaluation should focus on parent-infant relationship during childhood and neurodevelopmental long-term outcome.

## Conclusion

In settings where there is no home care available, early discharge of preterm infants with NTF embedded in a concept of family integrated care and rooming-in and close outpatient clinic follow-up appears to be safe and very well-accepted by parents. Most parents would recommend this practice to others, and stress levels of parents with NTF at home are low.

## Data Availability Statement

The raw data supporting the conclusions of this article will be made available by the authors, without undue reservation.

## Ethics Statement

The studies involving human participants were reviewed and approved by Medical Ethics Review Board of the National Baden-Wuerttemberg Medical Association, 70597 Stuttgart, Germany. The patients/participants provided their written informed consent to participate in this study.

## Author Contributions

RS and WM contributed to conception and design of the study. WM organized the database and performed the statistical analysis. RS wrote the first draft of the manuscript. WM and HE wrote sections of the manuscript. All authors contributed to manuscript revision, read, and approved the submitted version.

## Conflict of Interest

The authors declare that the research was conducted in the absence of any commercial or financial relationships that could be construed as a potential conflict of interest.
