# Supplementary material for: Safety and Parental Satisfaction With Early Discharge of Preterm Infants on Nasogastric Tube Feeding and Outpatient Clinic Follow-Up
Source: Front Pediatr. 2020 Aug 25;8:505. doi: 10.3389/fped.2020.00505 (PMC7491054; doi:10.3389/fped.2020.00505)
Supplement: Supplementary file 1 [file Table_1.DOCX]

Supplement 1. Original Questionnaire in German

1. Falls Sie sich noch erinnern können- was waren Ihre ersten Gedanken als Sie anfangs gehört haben, dass Sie Ihr Kind mit Magensonde nach Hause nehmen können?

2. Wie gut waren Sie zum Zeitpunkt der Entlassung darauf vorbereitet die Nahrung selbstständig zu sondieren? Bitte die zutreffende Zahl markieren

Gar nicht vorbereitet sehr gut vorbereitet

| 1 | 2 | 3 | 4 | 5 |
| --- | --- | --- | --- | --- |

3. Haben Sie zuhause irgendwelche Schwierigkeiten oder Probleme erlebt bei der Sondierung der Nahrung?

--- Ja, bitte beschreiben Sie die Probleme

--- Nein

4. Wie sehr hat das Sondieren der Nahrung Sie zuhause gestresst?

Gar nicht gestresst sehr gestresst

| 1 | 2 | 3 | 4 | 5 |
| --- | --- | --- | --- | --- |

5. Hatte es irgendeinen Vorteil für Sie und Ihre Familie, dass Sie Ihr Kind mit Magensonde nach Hause genommen haben?

--- Ja, bitte beschreiben Sie die Vorteile

--- Nein

6. Würden Sie anderen Familien in einer ähnlichen Situation empfehlen, die Sondierung zuhause durchzuführen?

--- JA --- Nein --- Vielleicht

7. Meinen Sie, Sie haben die richtige Entscheidung getroffen indem Sie Ihr Kind mit Magensonde nach Hause genommen haben oder wären Sie lieber im Krankenhaus geblieben bis Ihr Kind die Nahrung vollständig alleine trinken kann?

--- Froh mit Magensonde entlassen worden zu sein

--- Wäre gerne länger in der Klinik geblieben
